# More virological failure with lamivudine than emtricitabine in efavirenz and nevirapine regimens in the Dutch nationwide HIV Cohort

**DOI:** 10.7448/IAS.17.4.19491

**Published:** 2014-11-02

**Authors:** Casper Rokx, Azzania Fibriani, David van de Vijver, Annelies Verbon, Martin Schutten, Luuk Gras, Bart JA Rijnders

**Affiliations:** 1 Department of Internal Medicine, Erasmus University Medical Center, Rotterdam, Netherlands; 2 Department of Virology, Erasmus University Medical Center, Rotterdam, Netherlands; 3 On behalf of the ATHENA national observational cohort study. Dutch HIV Monitoring Foundation, Research Department, Amsterdam, Netherlands

## Abstract

**Introduction:**

Lamivudine (3TC) and emtricitabine (FTC) are considered interchangeable by HIV-1 guidelines in first-line tenofovir/efavirenz (TDF/EFV) and TDF/nevirapine (NVP) combination antiretroviral therapy (cART). Data from trials on equivalence of 3TC and FTC are inconsistent. We examined the effectiveness of 3TC and FTC in the national HIV cohort in the Netherlands.

**Material and Methods:**

Observational cohort study on cART naïve HIV-1 patients. Therapy was initiated as 3TC or FTC with TDF/EFV or TDF/NVP between 2002 and 2012. Patients with baseline resistance or prior cART experience were excluded. Main outcomes were Week 48 virological failure (VF) by on treatment analysis, time to HIV-RNA <400 copies/mL within 48 weeks and VF within 240 weeks after at least one HIV-RNA <400 copies/mL. Acquired resistance to reverse transcriptase was evaluated. Analyses were done by logistic regression and Cox proportional hazard models. Propensity score adjusted models and intention to treat evaluations were included as sensitivity analysis.

**Results:**

A total of 4836 patients initiated 3TC/TDF/EFV (*n*=546), FTC/TDF/EFV (*n*=3391), 3TC/TDF/NVP (*n*=207) or FTC/TDF/NVP (*n*=692). Ninety-six patients were excluded for baseline resistance or prior cART experience. By Week 48, VF proportions were higher for 3TC/TDF/EFV (10.8%) compared to FTC/TDF/EFV (3.6%) and for 3TC/TDF/NVP (27.0%) compared to FTC/TDF/NVP (11.0%). The multivariable adjusted odds ratio (OR) on VF was 1.78 (95% CI 1.11–2.84; *p*=0.016) with 3TC/TDF/EFV compared to FTC/TDF/EFV and 2.09 (95% CI 1.25–3.52; *p*=0.005) with 3TC/TDF/NVP compared to FTC/TDF/NVP. Propensity score adjusted models and intention to treat analyses showed comparable results. The time to virological suppression within 48 weeks was not influenced by using 3TC or FTC in cART. If HIV-RNA <400 copies/mL was achieved on initial cART first, no differences in VF within 240 weeks were observed between 3TC and FTC with TDF/EFV (*p*=0.090) or TDF/NVP (*P*=0.255). Patients failing 3TC-containing cART had higher median HIV-RNA at VF compared to FTC containing cART (*p*<0.001) and 89.8% had acquired resistance on 3TC compared to 81.2% on FTC.

**Conclusions:**

Including FTC in cART is associated with better virological responses compared to 3TC. As cost constraints may call for the use of generic 3TC, a well-powered randomized trial to confirm the presumed equivalence of 3TC and FTC is needed.

